# Changes in Erythrocytes in 88 Hyperthyroid Cats

**DOI:** 10.3390/ani14213136

**Published:** 2024-10-31

**Authors:** Olga Gójska-Zygner, Grzegorz Kotomski, Joanna Gajger, Luke J. Norbury, Wojciech Zygner

**Affiliations:** 1 LABROS Specjalistyczna Przychodnia Weterynaryjna, Św. Bonifacego 92, 02-940 Warsaw, Poland; olgazygner@yahoo.pl (O.G.-Z.); gkotomski@o2.pl (G.K.); joanna-gajger@wp.pl (J.G.); 2 Department of Biosciences and Food Technology, School of Science, STEM College, RMIT University, Bundoora, VIC 3083, Australia; luke.norbury2@rmit.edu.au; 3 Division of Parasitology and Parasitic Diseases, Department of Preclinical Sciences, Institute of Veterinary Medicine, Warsaw University of Life Sciences-SGGW, Ciszewskiego 8, 02-786 Warsaw, Poland

**Keywords:** feline hyperthyroidism, red blood cells, anaemia, erythrocytosis, microcytosis, macrocytosis

## Abstract

Background: There is only a limited number of studies that show alterations in erythrocytes in feline hyperthyroidism. Discrepancies between the findings of these studies may be caused by the presence of concurrent diseases and the use of various haematological analysers. Methods: This study analysed changes in red blood cells (RBCs) in 88 hyperthyroid cats without concurrent diseases, to identify associations between observed changes and to assess the influence of serum thyroxine (T4) concentration, cat age, and sex, on RBC changes. Results: Among the eighty-eight hyperthyroid cats, erythrocytosis was observed in twelve, anaemia in four, macrocytosis in two, and microcytosis in three. Three of the four cases of anaemia probably resulted from concurrent unrecognised disease. Interestingly, all cases of microcytosis were recognized in cats that had erythrocytosis. This study identified a moderate negative correlation between RBC count and mean corpuscular volume (MCV; r = −0.57, *p* < 0.001). Concluding, it appears that erythrocytosis may be a response to the development of microcytosis. The correlation between RBC count and MCV in hyperthyroid cats may result from the increased action of hepcidin and erythropoietin coupled with the decreased action of thyroid-stimulating hormone (TSH).

## Simple Summary

This study investigated changes in red blood cells (RBCs) in hyperthyroid cats and revealed that there was a moderate negative correlation between RBC count and mean corpuscular volume (MCV) in affected animals. This may result from the increased action of hepcidin and erythropoietin coupled with the decreased action of thyroid-stimulating hormone (TSH) in hyperthyroidism.

## 1. Introduction

Feline hyperthyroidism is a systemic disease caused by thyroid adenoma which causes increased secretion and action of thyroid hormones, i.e., thyroxine (T4) and triiodothyronine (T3). The disease is the most common endocrine disorder of middle-aged and older cats [1]. Since its discovery in cats in the late 1970s, disease prevalence has increased [1]. The highest prevalences have been observed in Ireland and Poland [2,3].

Shortly after the first report of feline hyperthyroidism in 1979, Peterson et al. [4] published a study of the pathological changes observed in 131 American hyperthyroid cats. That work included observations of erythrocyte changes, mainly erythrocytosis and macrocytosis. Similar results were obtained a decade later in 202 hyperthyroid cats from North America [5]. However, the initial European study of clinical pathology in a larger cohort of affected cats did not identify erythrocytosis as highly prevalent. Unfortunately, the authors of that research did not provide erythrocyte size measurements in cats with hyperthyroidism [6]. In 2021, another study from Europe identified neither erythrocytosis nor macrocytosis in 78 hyperthyroid cats; however, microcytosis was observed in 29.5% of cats [7], while two of eight hyperthyroid cats had microcytosis in a small study from North America [8].

Previous studies examining changes in erythrocytes in hyperthyroid cats showed discrepancies and had been undertaken exclusively in the UK and USA, and it was not clear whether reported erythrocyte changes resulted from hyperthyroidism or other concurrent diseases. Therefore, the authors conducted a retrospective study on changes in red blood cells in hyperthyroid cats from another European country. The objectives of this study were to identify changes in red blood cells in hyperthyroid cats without comorbidities from Poland, to find associations between observed changes and the T4 concentration, age, and gender of affected animals, and to identify any association between erythrocytosis or anaemia and macro- or microcytosis in feline hyperthyroidism. This was undertaken to better understand how erythrocyte changes manifest during feline hyperthyroidism.

## 2. Materials and Methods

### 2.1. Study Design

From January 2019 to December 2023 the medical records from 528 hyperthyroid cats were collected, these included: complete blood count (with microscopic examination of blood smear), serum sodium, total protein, albumin, urea, creatinine and total T4 concentration, abdominal ultrasonography, and chest X-ray. Blood and serum samples were collected during the initial visit to the clinic before any treatment. All blood samples were collected in the mornings. Additionally, faecal examination for the presence of occult blood (guaiac-based qualitative test) and intestinal parasitic infections, and immunochromatographic tests for the presence of FeLV antigen and antibodies against FIV were performed.

**Figure 1:**
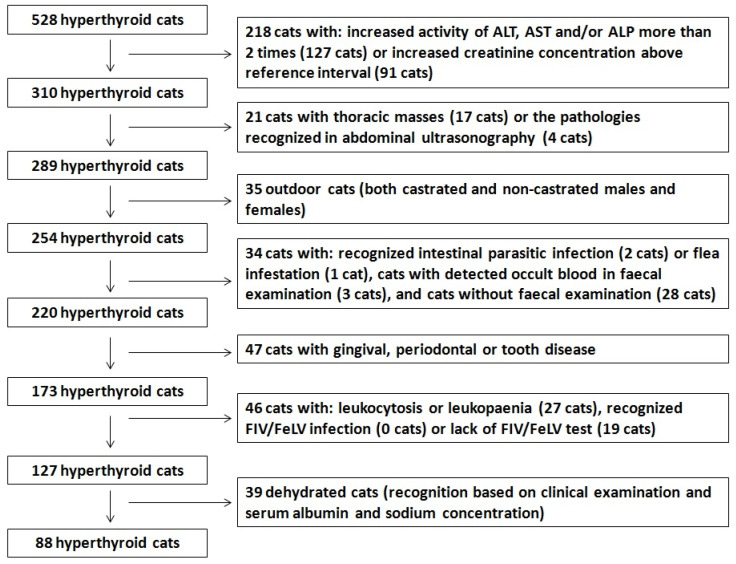
Chart showing exclusion of cats with concurrent diseases.

Diagnosis of hyperthyroidism was based on an increased serum total T4 concentration above 60 nmol/L (reference interval 10–30 nmol/L). Decrease of body weight, increased appetite, increased faecal volume, diarrhoea, vomiting, polyuria and polydipsia, and changes in behaviour were the main clinical signs observed in the cats. To exclude or limit various factors that might influence erythrocyte changes, cats with various conditions were excluded from the study (Figure 1). Cats with increased (more than two times the upper limit of the reference range) serum activity for alanine transaminase (ALT upper reference limit 91 IU/L), aspartate transaminase (AST upper reference limit 59 IU/L), or alkaline phosphatase (ALP upper reference limit 140 IU/L) were excluded from the study (127 cats). Cats with an increased concentration of serum creatinine above the reference interval (0.6–1.8 mg/dL) were also excluded (91 cats). Cats with pathologies recognized during ultrasonographic examination, including enlargement of the spleen or liver, masses in the spleen, liver, or alimentary tract (including significant wall thickening and enlargement of mesenteric lymph nodes), with renal tumours, or without ultrasonography were excluded from the study (65 cats). While fifty-six cats exhibited these pathologies and nine had no ultrasound examination at this step, only an additional four cats were excluded at this step, with sixty-one already excluded owing to increased serum creatinine or activities of liver enzyme. Moreover, at this step all cats with mediastinal or pulmonary mass recognized in X-ray examination were excluded from the study (17 cats). General enlargement of the lymph nodes was another exclusion criterion (recognized in 39 cats); however, all cats with this pathology were excluded previously due to thoracic masses or pathologies recognized in abdominal ultrasonography. Other exclusion criteria for cats remaining at this step (289 cats) were as follows: outdoor cats because of possible infections from other animals (both castrated and non-castrated males and females; thirty-five cats), recognized intestinal parasitic infection (two cats) or flea infestation (one cat), detection of occult blood in faecal examination (three cats), lack of faecal examination (twenty-eight cats), gingival, periodontal, or tooth disease (forty-seven cats), leukocytosis or leukopaenia (twenty-seven cats), recognized FIV/FeLV infection (zero cats) or lack of a test for FIV/FeLV infection (nineteen cats), and dehydration recognized basing on clinical examination (thirty-nine cats). All cats with increased total protein and albumin serum concentrations above reference intervals or cats with hypernatraemia were considered as dehydrated and included to the group of 39 dehydrated cats excluded from the study.

Eighty-eight castrated hyperthyroid cats were included in the study (42 males and 46 females). In this population 74 cats were non-pedigree. The remaining fourteen cats belonged to the following breeds: Main Coon (four cats), Persian cat (three cats), Siberian cat (two cats), Siamese cat (two cats), Russian Blue cat (one cat), Norwegian Forest cat (one cat), and Ragdoll (one cat). All eighty-eight cats included in the study were the sole cat in their households.

After diagnosis of hyperthyroidism, treatment with methimazole or thyroidectomy (if methimazole was ineffective) was recommended. Two months after restoration of a euthyroid state, complete blood count was advised.

### 2.2. Blood and Serum Analysis

Ethylenediamine tetraacetic acid (EDTA) was used as an anticoagulant. One millilitre blood samples were collected into two millilitre tubes coated with an amount of EDTA appropriate for one millilitre of blood. Samples were stored at room temperature and examined within 6 h. Complete blood counts were determined by a Sysmex XT-2000iV automatic haematological analyser (Sysmex, Kobe, Japan). Erythrocytosis was diagnosed in cats with red blood cell (RBC) counts higher than 11.2 × 10^6^/µL, while counts lower than 6.7 × 10^6^/µL indicated anaemia. Macrocytosis was diagnosed in cats with a mean corpuscular volume (MCV) higher than 49.3 fL, while microcytosis was defined as a MCV lower than 34.4 fL. Hypochromia was defined as mean corpuscular haemoglobin concentration (MCHC) lower than 19.1 mmol/L. The reference range for erythrocytes was based on the results of the study of Granat et al. [9]. Both macro- and microcytosis were confirmed by microscopic examination of blood smears.

Serum samples were obtained by centrifugation of blood samples collected in tubes without anticoagulant. The concentration of total T4 was determined in serum samples using an Immulite 2000 XPi clinical immunoassay analyser system (Siemens Healthcare Diagnostics Inc., Malvern, AL, USA). Serum albumin, total protein, urea and creatinine concentrations, and ALT, AST and ALP activities were determined using an Erba XL 640 clinical chemistry analyser (Erba, Mannheim, Germany). Serum sodium concentration was determined using a Medica Easy Electrolytes chemical analyser (Medica, Hague, The Netherlands).

### 2.3. Statistical Analysis

The results were analysed using the Statistica program version 13. Shapiro–Wilk’s W test was used for the estimation of normality in the distributions of cats’ age, T4 concentration, RBC count, haemoglobin (Hb) concentration, haematocrit (Hct), MCV, red blood cell distribution width—coefficient of variation (RDW-CV), and serum concentrations of sodium, albumin, total protein, urea, and creatinine in all 88 cats, within the 42 males and within the 46 females. Levene’s test for homogeneity of variances in males and females with normal distribution was used. Student’s *t*-test (normal distribution in both males and females, and lack of difference between variances) or Mann–Whitney U test (lack of normal distribution in at least one of the compared sex groups) were used to compare the age of cats, T4 concentration, RBC count, Hb concentration, Hct, MCV, and RDW-CV. Additionally, Yates corrected χ^2^ test was used to assess the influence of the sex of the cat on the development of erythrocytosis, anaemia, macrocytosis, or microcytosis. The Clopper–Pearson exact method was used to calculate 95% confidence intervals for the prevalence of erythrocytosis, anaemia, macrocytosis, and microcytosis in the 88 hyperthyroid cats. Pearson’s correlation coefficient (r; normal distribution) or Spearman’s rank correlation coefficient (R; lack of normal distribution) were used to calculate correlations between the age of cats, T4 concentration, RBC count, Hb concentration, Hct, MCV, and RDW-CV. In order to assess the influence of the hydration status of the cats on RBC count and MCV, Pearson’s or Spearman’s correlation coefficients (depending on normality of distribution) were used to calculate correlations between both RBC count and MCV and serum concentrations of total protein, albumin, sodium, and urea. Wilcoxon matched paired test was used to assess the influence of antithyroid therapy on RBC count and MCV. The value of *p* < 0.05 was considered statistically significant.

## 3. Results

### 3.1. Distribution of Variables in 88 Hyperthyroid Cats

The Shapiro–Wilk W test identified a normal distribution for age, RBC count, Hb concentration, Hct, MCV, and serum sodium and urea concentration in all 88 cats. A lack of normal distribution was observed in RDW-CV, MCHC, and the serum concentrations of T4, total protein, albumin, and creatinine in all cats (Table S1).

### 3.2. Distribution of Age, T4 Concentration and Erythrocyte Parameters in Males and Females, and Differences Between Sex Groups

A normal distribution for age of animals, RBC count, Hb concentration, Hct, and MCV was observed in both the group of 42 males and the group of 46 females. In both sex groups the T4 concentration and RDW-CV were not normally distributed (Supplementary Materials Tables S2 and S3). Levene’s test for homogeneity of variances with normal distribution of variables in males and females (age, RBC count, Hb concentration, Hct, MCV) showed homogenous variances. Student’s *t* test did not identify significant differences between age, RBC count, Hb concentration, Hct, and MCV of males and females (Table S4). Mann–Whitney U test showed a lack of differences between T4 concentration and RDW-CV in male and female cats (Table 1).

### 3.3. Prevalence of Erythrocytosis, Anaemia, Macro- and Microcytosis, Hypochromia, and Influence of Sex on These Changes

**Figure 2:**
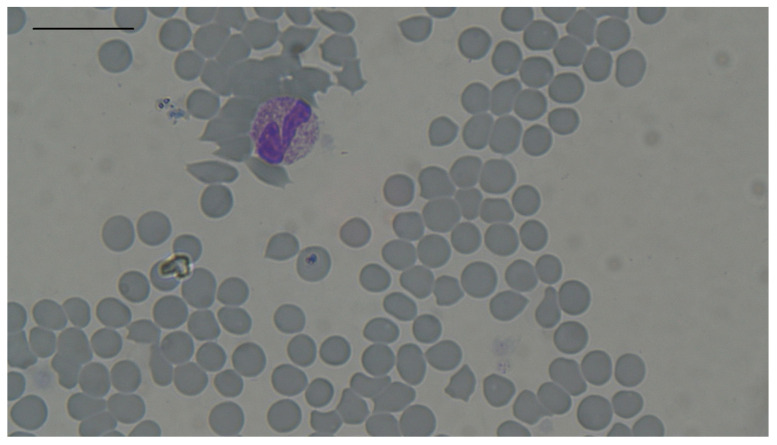
Macrocytosis in one of the hyperthyroid cats. Staining by May–Grunwald Giemsa method, magnification 100× *g*, scale bar 20 µm.

**Figure 3:**
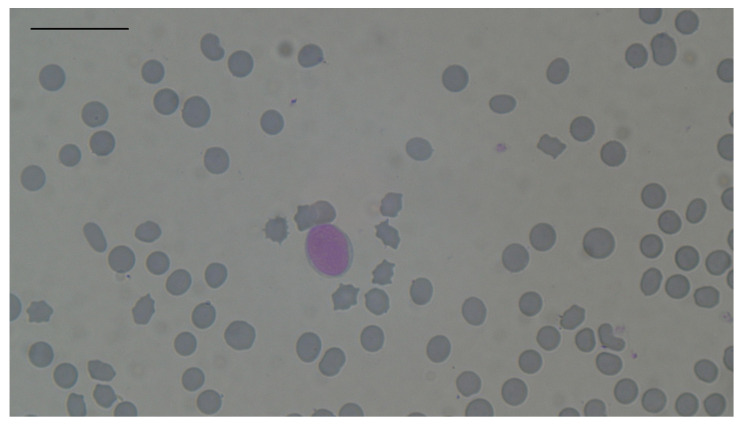
Microcytosis in one of the hyperthyroid cats. Staining by May–Grunwald Giemsa method, magnification 100× *g*, scale bar 20 µm.

Erythrocytosis was the most prevalent change observed in hyperthyroid cats, it was recognized in 12 out of 88 cats (13.64%; 95% CI: 7.25–22.61%). It was observed in five males and seven females. Anaemia was recognized in one male and three females (four out of eighty-eight cats; 4.55%; 95% CI: 1.25–11.23%). Macrocytosis was observed only in two females (two out of eighty-eight cats; 2.27%; 95% CI: 0.28–7.97%). Microcytosis was recognized in two males and one female (three out of eighty-eight cats; 3.41%; 95% CI: 0.71–9.64%). Hypochromia was recognized in six males and four females (ten out of eighty-eight cats; 11.36%; 95% CI: 5.59–19.91%). Interestingly, microcytosis was observed only in cats with erythrocytosis, and none of them had hypochromia. Both macro- and microcytosis were confirmed by microscopy (Figure 2 and Figure 3). Although RDW-CV was within reference intervals in all 88 cats, mild anisocytosis was observed during microscopic examination in all cats. Yates corrected χ^2^ test did not identify sex as having an influence on the development of erythrocytosis, anaemia, or macro- or microcytosis (Table 2).

### 3.4. Influence of Age and T4 Concentration on RBC Count, Hb Concentration, Hct, MCV and RDW-CV in 88 Hyperthyroid Cats

There were no correlations between age (Pearson’s correlations) and RBC count, Hb concentration, Hct, and MCV. Correlations between T4 concentration (Spearman’s rank correlation), and RBC count, Hb concentration, and MCV were statistically insignificant. No correlation (Spearman’s rank correlation) was observed between T4 concentration and Hct. Spearman’s rank correlation coefficients, assessing correlations between RDW-CV and both the age of cats and T4 concentration, showed a lack of correlations (Table 3). Spearman’s rank correlation between the age of cats and T4 concentration was statistically insignificant (R = 0.166, *p* = 0.121).

### 3.5. Correlations Between Erythrocyte Parameters in 88 Hyperthyroid CATS

**Figure 4:**
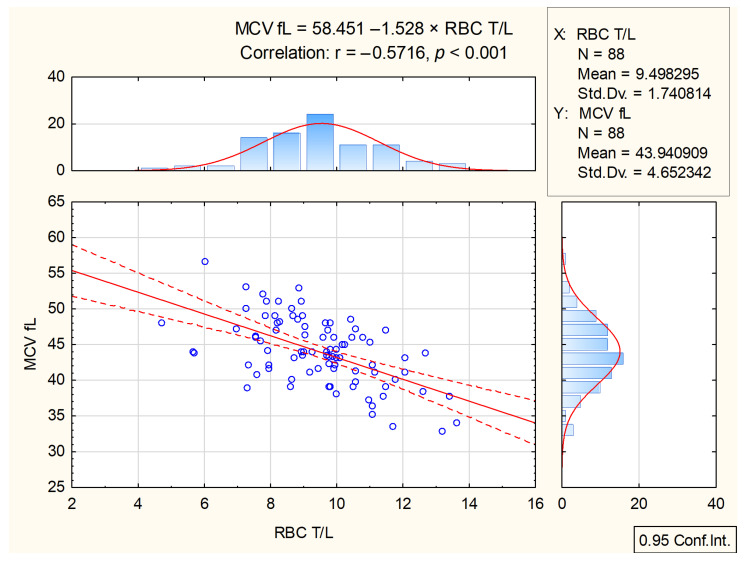
Correlation between MCV and RBC count in 88 hyperthyroid cats (solid line) with 95% confidence interval (dotted line); circles—cases of hyperthyroid cats. Horizontal (top) histogram—distribution of RBC count in 88 hyperthyroid cats, vertical (side) histogram—distribution of MCV in 88 hyperthyroid cats.

Correlations between RBC count, Hb concentration, Hct, and MCV were identified using Pearson’s correlation coefficient. However, there was no correlation between MCV and Hct, and the correlation between MCV and Hb concentration was statistically insignificant (Table S5). Three of the erythrocyte parameters (RBC count, Hb concentration, and Hct) exhibited clear and strong positive correlations, indicating strict association with each other (Figure S1). Of particular interest was the correlation between RBC count and MCV, which was a moderate, negative, statistically significant correlation (Figure 4). No correlation was observed between RDW-CV and either RBC count or Hb concentration, while any correlation with MCV and Hct was not statistically significant (Spearman’s).

### 3.6. Correlations of RBC Count and MCV with Serum Concentrations of Sodium, Total Protein, Albumin, Urea and Creatinine in 88 Hyperthyroid Cats

There were no statistically significant correlations between RBC count and the serum concentrations of sodium or total protein (Table 4). Additionally, no correlations between RBC count and either albumin or creatinine concentrations were observed. There was a weak negative correlation between RBC count and serum urea concentration.

No correlations between MCV and the concentrations of serum sodium, total protein, albumin, or creatinine were observed. However, there was weak positive correlation between MCV and the serum urea concentration (Table 4).

### 3.7. Influence of Antithyroid Therapy on RBC Count and MCV

The results of haematological examination following restoration of a euthyroid state were obtained from only nineteen of the eighty-eight cats included in the study. All nineteen cats were treated only with methimazole. Eighteen of the nineteen cats that achieved restoration of a euthyroid state had both RBC count and MCV within reference intervals both before treatment and after restoration of a euthyroid state. Only one of these nineteen cats had microcytosis and erythrocytosis before treatment. After restoration of a euthyroid state, both these parameters increased in this cat. Changes in RBC count and MCV in these 19 cats compared to baseline (pre-treatment values) were as follows. Increased RBC counts were observed in eight cats, while decreased RBC counts were detected in eleven cats. Increased MCV was noted in thirteen cats, while decreased MCV was observed in six cats. Wilcoxon matched paired test analysis identified no influence of antithyroid therapy on either RBC count (Z value 0.443; *p* 0.658) or MCV (Z value 1.569; *p* 0.116) in the nineteen cats two months after restoration of a euthyroid state.

## 4. Discussion

### 4.1. Lack of Influence of Sex, Age, and T4 Concentration on Erythrocyte Parameters

Our study showed a lack of influence of sex and age on changes in red blood cells in castrated hyperthyroid cats. This differs from results observed in hyperthyroid humans, in which the risk for development of anaemia is higher in women [10]. The lack of difference observed between males and females in our study may result from the fact that all cats were castrated. However, the lack of influence of age on changes in erythrocytes also contrasts with the study of human hyperthyroidism. In a meta-analysis of cohorts from various continents, Wopereis et al. [10] showed that increasing age was associated with an increased risk of anaemia in hyperthyroid human patients. However, that work included results from studies which included patients with concurrent diseases. In our study we tried to exclude all hyperthyroid cats with co-morbidities. Moreover, Wopereis et al. [10] incorporated results from studies on various thyroid disorders in their research. However, feline hyperthyroidism shares similarities with only one of the forms of human hyperthyroidism, i.e., toxic multinodular goitre, and there is no feline disease similar to immune-mediated Graves’ disease, which is the most common cause of human hyperthyroidism [11,12].

Similar to the findings in cats by Gil-Morales et al. [7], no correlation between the concentration of T4 and MCV was identified in our study. Moreover, a lack of correlation between the T4 concentration and other red blood cell parameters such as RBC count, Hb concentration, Hct, or RDW-CV was observed. This lack of correlations may be partially associated with fluctuations in T4 levels in hyperthyroid cats [13]. Gil-Morales et al. [7] showed that hyperthyroidism severity may be associated with the prevalence of microcytosis in affected cats. Microcytosis was observed in 23 out of 78 cats and was diagnosed more frequently in cats suffering from moderate (total T4 concentration 125–250 nmol/L) and severe (total T4 concentration > 250 nmol/L) hyperthyroidism compared to the mild form of the disease (total T4 concentration 60.1–124.9 nmol/L) [7]. No such comparison was possible in this study as only three cats had recognized microcytosis. Moreover, assessing hyperthyroidism severity using the criteria of Gil-Morales et al. [7], cats in this study were only classified to the mild or moderate groups.

### 4.2. Microcytosis

In cats, microcytosis is mainly caused by iron deficiency. In healthy cats it may occur in kittens with an iron-deficient milk diet [14]. Moreover, most Abissynian cats have MCV below the reference interval [15].

It should be mentioned that Gil-Morales et al. [7] considered all cats with a MCV lower than 41.3 fL as microcytic. In our study, microcytosis was recognized in cats with a MCV lower than 34.4 fL. This difference stems from the analysers used; Gil-Morales et al. [7] used a Siemens haematologic analyser, in which the MCV reference interval lower limit was established by the analyser manufacturer as 41.3 fL. Alternatively, our study utilised a Sysmex haematologic analyser for which Granat et al. [9] established 34.4 fL as the low limit for the MCV reference interval. This difference in haematologic analysers (and reference intervals) may account for some of the disparity between the results of this study and the earlier work of Gil-Morales et al. [7]. Another explanation for differences may stem from variations in the design of the studies. Gil-Morales et al. [7] included cats with various co-morbidities, while we tried to exclude all cats with concurrent diseases. This is probably not completely possible in older cats but may limit the number of such animals. Thus, the absence of hyperthyroid cats with other diseases (or the low prevalence of co-morbidities) may be another explanation for the observed low prevalence of microcytosis in this study. Nevertheless, the detection of microcytosis in this work aligns more closely to the findings from the UK obtained by Gil-Morales et al. [7], in contrast to the results of the initial studies from the USA where none of the hyperthyroid cats had microcytosis [4,5]. We have only identified a single work from the USA which diagnosed microcytosis in two hyperthyroid cats; however, only eight cats were included in that research [8]. It is interesting that microcytosis was not diagnosed in feline hyperthyroidism in earlier studies [4,5] but is observed in hyperthyroid cats in more recent studies in both Europe and North America [7,8]. The improved accuracy of haematological analysers may be a possible explanation for this difference.

As mentioned by Gil-Morales et al. [7] microcytosis has also been observed in humans with hyperthyroidism. How et al. [16] described the decrease in MCV as the most significant finding in the haematological data in untreated non-anaemic hyperthyroid humans. Kawa et al. [17] recognized microcytosis in 42% of untreated hyperthyroid human patients, despite observing an increased mean MCV in the hyperthyroid cohort. Both Kawa et al. [17] and Gil-Morales et al. [7] suggested restriction of iron in haematopoiesis as the reason for microcytosis in human and feline hyperthyroidism, respectively. This supposition may be confirmed by the detection of hypochromia in 10 cats in the presented study. Surprisingly, none of the microcytic cats had decreased MCHC. However, according to Abrams-Ogg [14] hypochromia is often not present in cats with iron deficiency.

The action of hepcidin (a peptide hormone with antimicrobial properties secreted by the liver) may explain the occurrence of microcytosis in hyperthyroid cats. Hepcidin plays a role in inhibiting iron absorption from the duodenum and iron mobilisation from macrophages and hepatocytes [18]. It has been shown that pro-inflammatory cytokine interleukin 6 (IL-6) is one of the main triggers for increased hepcidin expression, and increased IL-6 serum levels are observed in hyperthyroid human patients with both Graves’ disease and toxic adenoma [19,20,21,22]. Moreover, in an in vitro study, Fischli et al. [23] showed that T3 led to increased hepcidin mRNA expression in the human hepatoma HepG2 cell line. It has also been demonstrated that serum hepcidin concentration significantly decreases in human patients with Graves’ disease after restoration of euthyroidism, in comparison to levels before treatment [23,24]. Thus, it is possible that microcytosis in feline hyperthyroidism may result from increased levels of hepcidin in affected cats. High hepcidin concentration has been observed in cats with chronic kidney disease, including one cat with hyperthyroidism [25]. However, the study authors note that the serum concentration of hepcidin in feline hyperthyroidism has not been studied, and further research on hepcidin levels and its association with changes in red blood cells in hyperthyroid cats is needed.

Gil-Morales et al. [7] also mentioned that hyponatraemia can be another explanation for an observed decrease in MCV. This results from inaccuracy in the measurement of the MCV caused by a hypoosmolar environment [26]. However, in this study, similar to Gil-Morales et al. [7], none of the cats had a decreased serum sodium concentration. Moreover, our study surprisingly showed a lack of correlation between the sodium concentration and MCV. This could stem from the influence of other factors, possibly hepcidin action, affecting the association between MCV and sodium concentration. Thus, microcytosis in this study did not result from changes in sodium concentration.

It is worth mentioning that hypertension might also contribute to the development of microcytosis in hyperthyroid cats. Reed and Hendley [27] showed an association between hypertension and decreased MCV in spontaneously hypertensive rats. As a significant percentage of hyperthyroid cats have hypertension [6], an association between blood pressure and MCV cannot be excluded in feline hyperthyroidism. On the other hand, Peterson et al. [4] and Broussard et al. [5] did not report a decrease in MCV in their studies of hyperthyroid cats, despite describing clinical signs indicative of hypertension.

### 4.3. Macrocytosis

Macrocytosis was another change in red blood cell size observed in our study. This finding contrasts with the results of Gil-Morales et al. [7] but exhibits similarities with the results from the early American studies [4,5]. However, macrocytosis was recognized only in two cats in our study, while Peterson et al. [4] observed an increased MCV in 58 out of 131 hyperthyroid cats. It is plausible that excluding or limiting cats with concurrent diseases in our study could be the main reason for the different findings between this research and the earlier work of Peterson et al. [4]. As mentioned above, a newer and more accurate generation of haematological analysers could also explain this difference. On the other hand, Gil-Morales et al. [7] did not detect macrocytosis in the cats from their study. While this difference could be attributed to variations in MCV reference intervals for different haematological analysers (Siemens vs. Sysmex), it is noteworthy that in both cats with macrocytosis from our study, MCV was also higher than the reference interval upper limit from the previous research, i.e., 52.6 fL [7]. The action of thyroid hormones could be an explanation for the cause of macrocytosis observed in this study. As mentioned above, increased MCV has been observed in humans with Graves’ disease [17]. According to Kawa et al. [17] an excess of thyroid hormones may lead to bone marrow stimulation and the release of immature erythrocytes into peripheral blood. This action of thyroid hormones also results in erythrocytosis [17]. However, in our study neither of the two cats with macrocytosis had concurrent erythrocytosis, and, as per the rest of the cats in this study, both had RDW-CV within reference intervals. Moreover, a moderate negative correlation between MCV and RBC count was observed. Thus, it appears the macrocytosis observed in our study was not the result of bone marrow stimulation by thyroid hormones.

Cobalamin and/or folate deficiency in hyperthyroid cats could also explain the macrocytosis observed in this study. Decreased serum concentrations of cobalamin and folate have been observed in hyperthyroid cats in previous studies [28,29]. It has been suggested that low serum levels of these vitamins in feline hyperthyroidism may result from decreased absorption, increased excretion, or increased utilisation [28,29]. In humans both deficiencies may lead to macrocytic anaemia [30]. In one study, hypocobalaminaemia in cats was also associated with increased MCV [31]. However, in the presented study only one of the two cats with macrocytosis had anaemia, and it seems plausible this cat might have cobalamin and/or folate deficiency. The second cat, however, had neither anaemia nor erythrocytosis. Thus, it seems probable that an undetected co-morbidity was the reason for macrocytosis in this cat.

### 4.4. Anaemia

Both increases and decreases in RBC count were observed in this study, leading to erythrocytosis or anaemia, respectively. This is similar to previous studies examining human and feline hyperthyroidism, with both anaemia and erythrocytosis observed [4,32,33]. In our work anaemia was not prevalent; this result is similar to the results of other studies on feline hyperthyroidism [4,6,7]. A decreased RBC count was observed only in four cats. In one cat anaemia was macrocytic. This may suggest cobalamin or folate deficiency. Cook et al. [28] observed lower RBC counts in hyperthyroid hypocobalaminaemic cats compared to hyperthyroid cats with normocobalaminaemia. However, this difference was statistically insignificant, and none of the cats with decreased serum cobalamin concentration were anaemic in that study [28].

Anaemia was normocytic in the other three cats in our study, and in these cats anaemia was probably not associated with cobalamin or folate deficiency. Gil-Morales et al. [7] reported similar results, observing anaemia in one hyperthyroid normocytic cat and two cats with microcytosis. Concurrent illness was suspected as the reason for anaemia in the hyperthyroid normocytic cat [7]. In our study we cannot exclude concurrent disease as the cause of anaemia in the three hyperthyroid normocytic cats. Sehgal et al. [34] suggested that treatment with β blockers may be one of the reasons for development of anaemia in hyperthyroid humans with unknown cause for this complication. However, cats from this study were not treated with any drugs before blood collection. Microcytic anaemia was observed by Gil-Morales et al. [7] in two cats. According to Sehgal et al. [34] this form of anaemia can be caused by progressing thyrotoxicosis leading to ineffective erythropoiesis. In our work none of the cats with microcytosis had anaemia.

Noteworthy observations have been made in pigs with experimental hyperthyroidism. Treatment with a daily dose of 20 μg/kg of levothyroxine for 8 weeks led to the mean RBC count decreasing from 6.215 × 10^6^/µL before experiment to 5.6 × 10^6^/µL. Subsequently four weeks after the cessation of levothyroxine treatment the RBC count increased to 6.76 × 10^6^/µL [35]. Unfortunately, the authors did not report on MCV changes in the experiment animals [35]; nevertheless, the study demonstrated hyperthyroidism can influence RBC count in affected animals. However, the exact mechanism underlying the development of anaemia in hyperthyroidism remains unknown.

### 4.5. Erythrocytosis

Erythrocytosis was the most prevalent change observed in this study. In comparison with our other results, this observation is similar to the results of the initial American study, in which Peterson et al. [4] recognized increased RBC counts in 27 out of 131 hyperthyroid cats. However, the prevalence of erythrocytosis was lower in our research. On the other hand, our result is in stark contrast to the results of the British studies [6,7]. Thoday and Mooney [6] detected an increased RBC count in only one out of fifty-seven hyperthyroid animals, and Gil-Morales et al. [7] did not observe erythrocytosis in any of 78 cats with hyperthyroidism. The influence of concurrent illness on the RBC count of the cats in the previous British studies or undetected comorbidity in our study seems to be possible reason for this discrepancy.

Erythrocytosis has also been observed in human hyperthyroidism [17,36]. Liu et al. [37] observed correlations between the serum level of hypoxia-inducible factor-1α (HIF-1α) and the concentrations of T3, T4, and erythropoietin (EPO) in hyperthyroid patients with Graves’ disease. Those authors suggested that erythrocytosis in hyperthyroidism may result from the activation of HIF-1α, leading to increased expression of the EPO gene [37]. Otto and Fandrey [38] showed that T3 leads to activation of the Thyroid Hormone Receptor β/Retinoid X Receptor α (TRβ/RXRα) heterodimer, and the result of this activation is stimulation of hepatic leukaemia factor (HLF). HLF then leads to increased expression of HIF-1α, the target gene of which is EPO [38,39]. Moreover, it has been shown in rats that T3 leads to increased expression of the Sonic hedgehog (*SHH*) gene [40]. Perry et al. [41] showed in an in vitro study that the protein encoded by *SHH* induces bone morphogenetic protein 4 (BMP-4) dependent expansion of stress erythroid burst-forming units (BFU-E) in mice. The BFU-E population increases in a BMP-4 dependent pathway in the spleen and liver during stress erythropoiesis, which is a part of the inflammatory state and a response to anaemia and tissue hypoxia [42]. Sivertsson et al. [43] showed that in rats T3 can induce kidney tissue hypoxia through increased oxygen metabolism. Thus, it seems possible that similar mechanisms (T3 → TRβ/RXRα → HLF → HIF-1α → EPO and T3 → SHH → BMP-4 → BFU-E) may lead to increased erythropoiesis in human and feline hyperthyroidism and may explain the presence of erythrocytosis observed in this disease.

### 4.6. Correlations Between Erythrocyte Parameters; Moderate Negative Correlation Between RBC Count and MCV

This study identified correlations between the RBC count, Hb concentration, Hct, and MCV. Positive correlations between the RBC count, Hb concentration, and Hct are expected, as these parameters are strongly associated. However, the negative correlation between the RBC count and MCV is somewhat unexpected, and the authors of this study find this result particularly intriguing. As mentioned above, all cases of microcytosis were observed in cats with erythrocytosis, and of the two cats with macrocytosis, one had anaemia. It appears erythrocytosis may be a response to the development of microcytosis and that the changes in cell size and RBC count may result from the action of both hepcidin and erythropoietin.

In this context, the action of cyclin D3 and the interactions between cyclin D3, T3, and thyroid-stimulating hormone (TSH) should be also mentioned. Cyclin D3 is a member of the cyclin protein family which controls the cell cycle [44]. It has been shown that deletion of cyclin D3 causes a reduction in cell divisions in erythropoiesis and results in increased MCV [45]. Sterle et al. [46] identified increased cyclin D3 expression in tumours from hyperthyroid mice. In a separate study, Meng et al. [47] showed that cyclin D3 leads to inhibition of T3 action in intestinal epithelia. While in research on human thyrocytes, Paternot et al. [48] showed that TSH activates cyclin D3. It is well known that thyrocytes are target cells for TSH; however, TSH receptors have also been found in other cells, including bone marrow cells and also erythrocytes [49,50]. However, in both feline and human hyperthyroidism the levels of TSH are low, due to a negative feedback loop [51,52]. In hyperthyroid cats, the low concentration of TSH may lead to reduced activation of cyclin D3 in erythropoiesis. This supposition is supported by the study of Montagnana et al. [53] who observed a positive but weak correlation between TSH level and MCV in over 3000 women referred to the clinical laboratory in Verona for routine blood testing. However, according to the authors’ knowledge, the influence of TSH on cyclin D3 in erythropoiesis in hyperthyroidism has not been investigated, either in humans or animals. This leads to the question: does the low level of TSH in hyperthyroid cats influence erythropoiesis, especially the MCV and RBC count? It seems that the relationship between cyclin D3, TSH, and T3, and inverse correlation between MCV and RBC count observed in this study, may be associated with a lack of cyclin D3 activation, inhibition of cyclin D3, or inhibition of its gene expression. On the other hand, in humans T3 has been linked with improved erythroblast differentiation and maturation of RBCs including decreasing size of RBCs [54]. Moreover, the relationship between cyclin D3, TSH, and T3 may be altered through the action of deiodinases, which, depending on the type, can activate or deactivate thyroid hormones [55]. Further study of the interactions between T3, TSH, cyclin D3, and RBC count and MCV in hyperthyroid patients can help resolve the problem presented here.

It should also be mentioned that hydration status and acid-base disturbances may have an influence on any increase or decrease of the MCV [56]. However, in this research, there was either a lack of correlation or statistically insignificant correlations between MCV and the serum concentrations of sodium, albumin, total protein, and creatinine. Although the creatinine concentration was within reference interval in all cats, and there was no correlation between MCV and creatinine levels, a weak significant correlation between serum urea concentration and MCV was observed in this study. This might be partially associated with the beginning of azotaemia development in hyperthyroid cats. Such changes have been observed in rats with unilateral renal agenesis; compared to control healthy rats, the urea concentration and MCV were higher, and creatinine level was lower in affected rats [57]. Similar to the correlations observed for MCV, the lack of correlations or absence of significant correlations between RBC count and serum concentrations of sodium, albumin, total protein, and creatinine indicate that the erythrocytosis observed in this study did not result from dehydration. Therefore, it was not relative erythrocytosis, but a true increase in RBC mass observed in hyperthyroid cats.

### 4.7. Limitations

The main limitation of this study was the lack of molecular examination of the cats for the presence of *Mycoplasma* and *Bartonella* infections, both of which may lead to anaemia in cats [58,59]. However, the owners of the study cats declared that none had ever had a flea (or tick) infestation, and these parasites are considered the main vectors of both pathogens in cats. Moreover, none of the cats included in the study were outdoor cats or had a history of fighting with other cats, as all the cats were the only animals in their household. It should be emphasised here that feline hyperthyroidism, as a systemic disease of middle-aged and older cats, may lead to other pathologies. Moreover, older cats might have concurrent diseases which were not recognized in this report. It is impossible to completely exclude all co-morbidities in older hyperthyroid cats, but the occurrence of other diseases was limited in this study.

Another limitation of the study was the low number of cats that provided follow-up haematological data two months after restoration of an euthyroid state. Both increases and decreases to RBC count and MCV were noted in these nineteen cats after antithyroid therapy. While a lack of statistical significance was observed, the small sample size impacted the statistical analysis and restricted the conclusions that could be drawn from this data. This limitation highlights an area for consideration in future studies that would provide an opportunity to explore the resolution of blood changes following the restoration of euthyroidism. It is worth noting that one of the nineteen euthyroid cats with previously observed microcytosis and erythrocytosis still had both pathologies after treatment, but MCV slightly increased (from 32.8 fL to 33.1 fL). Gil-Morales et al. [7] also observed microcytosis in two out of eight hyperthyroid cats long term after radioiodine therapy; however, microcytosis resolved in most of the previously microcytic cats (six out eight) 54 to 605 days after radioiodine therapy [7].

Other limitations of the study are that it is retrospective, we did not determine iron level or total iron-binding capacity, and did not exclude cats with cardiac disease.

It should be mentioned that we have used simplified definitions of erythrocytosis and anaemia, which were based only on RBC count. This was done for practical reasons and facilitated the classification of the cats with anaemia or erythrocytosis. According to various sources erythrocytosis can be defined as an increase of RBC count, or an increase of RBC count and Hct, or as an increase of RBC count, Hct, and Hb concentration [60,61,62]. In practice various combinations of these changes are observed. For this reason we used the simplest definition of erythrocytosis, i.e., increased RBC count. The same simplification was made with the definition of anaemia used in this study.

## 5. Conclusions

In most cases of feline hyperthyroidism anaemia probably results from concurrent disease. However, in rare cases anaemia may occur in hyperthyroid animals as the result of thyroid hormone action and can possibly be associated with Heinz body formation [63]. Erythrocytosis is more prevalent than anaemia in feline hyperthyroidism. It is caused by thyroid hormone action and is associated with microcytosis. These two changes (erythrocytosis and microcytosis) together may result from indirect action of thyroid hormones. According to the authors’ knowledge this is the first report of a negative correlation between RBC count and MCV in feline hyperthyroidism. This study provides news insights into erythrocyte changes during feline hyperthyroidism, and although this currently has limited practical significance, we believe that in future this knowledge may be useful, especially in regard to hepcidin action (and potential for use of hepcidin antagonists) in hyperthyroid cats.

## Figures and Tables

**Table 1:** Results of Mann–Whitney U test comparing T4 concentration and RDW-CV in male and female cats.

Variable	Males			Females		U	*p*
	Med.	25–75th%		Med.	25–75th%		
T4 nmol/L	81.5		69–109	94.5	73–143	790	0.143
RDW-CV %	23.2		20.9–24.3	23.25	20.4–24.1	924.5	0.732

Med.: median; 25–75th%: interval between twenty fifth and seventy fifth percentile; U a value of U; *p*: a value of *p*; T4: thyroxine concentration; RDW-CV: red blood cell distribution width—coefficient of variation.

**Table 2:** Results of Yates corrected χ^2^ test on the influence of sex on the development of erythrocytosis, anaemia, and macro- or microcytosis.

RBC Change	χ^2^	*p*	Number of Hyperthyroid Cats
Erythrocytosis	0.05	0.824	36 males with normal RBC count, 5 males with erythrocytosis
			36 females with normal RBC count, 7 females with erythrocytosis
Anaemia	0.21	0.646	36 males with normal RBC count, 1 male with anaemia
			36 females with normal RBC count, 3 females with anaemia
Macrocytosis	0.40	0.527	40 males with normal MCV, 0 males with increased MCV
			43 females with normal MCV, 2 females with increased MCV
Microcytosis	0.00	0.967	40 males with normal MCV, 2 males with decreased MCV
			43 females with normal MCV, 1 female with decreased MCV

χ^2^: a value of chi square; *p*: a value of *p* in Yates corrected χ^2^ test; RBC: red blood cell; MCV: mean corpuscular volume.

**Table 3:** Correlations of age of cats and T4 concentration with RBC count, Hb concentration, Hct, MCV, and RDW-CV.

Correlations	Age of Cats		T4 Concentration	
	r	*p*	R	*p*
RBC count	0.0056	0.959	−0.1142	0.289
Hb concentration	0.0143	0.895	−0.141	0.190
Hct	0.0372	0.731	0.019	0.858
MCV	0.0698	0.518	0.136	0.207
RDW-CV	−0.059 *	0.584	−0.087	0.420

R: Pearson’s correlation coefficient; R: Spearman’s rank correlation coefficient; T4: thyroxine; RBC: red blood cell count; Hb: haemoglobin concentration; Hct: haematocrit; MCV: mean corpuscular volume; RDW-CV: red blood cell distribution width—coefficient of variation; * non-parametric correlation between RDW-CV and age of cats (Spearman’s rank correlation).

**Table 4:** RBC count and MCV correlations with serum concentrations of sodium, total protein, albumin, urea, and creatinine in 88 hyperthyroid cats.

Correlation	Sodium	Total Protein	Albumin	Urea	Creatinine
RBC	r: 0.198, *p*: 0.064	R: 0.159, *p*: 0.139	R: 0.032, *p*: 0.767	r: −0.249, *p*: 0.019	R: 0.019, *p*: 0.857
MCV	r: −0.074, *p*: 0.491	R: −0.085, *p*: 0.432	R: 0.062, *p*: 0.562	r: 0.257, *p*: 0.016	R: 0.023, *p*: 0.831

RBC: red blood cell count; MCV: mean corpuscular volume; r: Pearson’s correlation coefficient; R: Spearman’s rank correlation coefficient; *p*: a value of *p*.

## Data Availability

The data presented in this study are available in this paper.

## Supplementary Materials

The following supporting information can be downloaded at: https://www.mdpi.com/article/10.3390/ani14213136/s1, Figure S1: 3D surface function showing correlations between haematocrit, haemoglobin concentration and red blood cell count in 88 hyperthyroid cats (blue circles—cases; colour scale from green to brown for haematocrit); Table S1: Statistics and results of Shapiro–Wilk W test in 88 hyperthyroid cats; Table S2: Statistics and results of Shapiro–Wilk W test in 42 hyperthyroid male cats; Table S3: Statistics and results of Shapiro–Wilk W test in 46 hyperthyroid female cats; Table S4: Results of Levene’s test for homogeneity of variances in males and females (age, RBC count, Hb concentration, Hct, and MCV), and results of Student’s *t* test comparing age, RBC count, Hb concentration, Hct and MCV between male and female cats; Table S5: Correlations between erythrocyte parameters in 88 hyperthyroid cats; Table S6: Values of red blood cell count and mean corpuscular volume in the cats with recognized changes; Raw data.
