# Supplementary material for: Changes in Erythrocytes in 88 Hyperthyroid Cats
Source: Animals (Basel). 2024 Oct 31;14(21):3136. doi: 10.3390/ani14213136 (PMC11545726; doi:10.3390/ani14213136)
Supplement: Supplementary file 1 [file animals-14-03136-s001.zip › Supplementary tables and Figures.pdf]

Table S1. Statistics and results of Shapiro-Wilk W test in 88 hyperthyroid cats.

	Mean	S.D.	Med.	25th%	75th%	Min.	Max.	W test result		Ref. int. *
								W	p	
Age in years	12.78	2.96	13	11	15	4	19	0.98	0.181	–
T4 nmol/L	106.5	49.9	86.5	69	130	61	240	0.81	0.000	10 – 30
RBC × 10^6/μL	9.50	1.74	9.70	8.245	10.6	4.72	13.62	0.99	0.812	6.7 – 11.2
Hb mmol/L	8.42	1.40	8.405	7.695	9.305	4.16	11.43	0.98	0.309	6.15 – 10.29
Hct L/L	0.412	0.062	0.42	0.38	0.455	0.23	0.56	0.97	0.118	0.29 – 0.51
MCV fL	43.94	4.65	43.95	41	47.15	32.8	56.6	0.99	0.978	34.4 – 49.3
RDW-CV %	22.29	2.76	23.2	20.65	24.1	13.4	27.5	0.90	0.000	19.7 – 29.3
MCHC mmol/L	20.46	1.44	20.2	19.4	21.25	17.1	24.8	0.95	0.003	19.1 – 24.3
Total protein g/L	71.65	4.52	71	68	75	64	80	0.95	0.003	60 – 80
Albumin g/L	34.37	2.84	34	32	37	28	40	0.96	0.020	27 – 39
Creatinine mg/dL	1.29	0.289	1.3	1.05	1.5	0.8	1.8	0.93	0.000	0.6 – 1.8
Urea mg/dL	65.04	18.57	64	49.6	77.5	26	99	0.98	0.126	25 – 70
Sodium mmol/L	152.0	2.88	152.45	149.85	154.05	145.6	157.1	0.97	0.073	145 – 158

* Haematological reference intervals based on the results of the study of Granat et al. [9]; biochemical reverence intervals according to a commercial veterinary laboratory from Warsaw in Poland (Lab-Wet); S.D.: standard deviation; Med.: median; 25th%: twenty fifth percentile; 75th%: seventy fifth percentile; Min.: minimal value; Max.: maximal value; W: a value of W in Shapiro–Wilk's W test; p: a value of p in Shapiro–Wilk's W test; Ref. int.: reference interval; T4: thyroxine concentration; RBC: red blood cell count; Hb: haemoglobin concentration; Hct: haematocrit; MCV: mean corpuscular volume; RDW-CV: red blood cell distribution width - coefficient of variation; MCHC: mean corpuscular haemoglobin concentration.

Table S2. Statistics and results of Shapiro-Wilk W test in 42 hyperthyroid male cats.

	Mean	S.D.	Med.	25th%	75th%	Min.	Max.	W test result		Ref. int. *
								W	p	
Age in years	12.95	2.89	13	11	15	7	18	0.97	0.432	–
T4 nmol/L	102	52.7	81.5	69	109	61	240	0.73	0.000	10 – 30
RBC × 10^6/μL	9.39	1.58	9.13	8.27	10.2	5.68	13.62	0.98	0.870	6.7 – 11.2
Hb mmol/L	8.39	1.31	8.33	7.51	9.2	5.03	10.71	0.98	0.840	6.15 – 10.29
Hct L/L	0.407	0.054	0.41	0.38	0.44	0.25	0.54	0.97	0.362	0.29 – 0.51
MCV fL	43.74	4.44	43.35	41	47	33.5	52	0.98	0.645	34.4 – 49.3
RDW-CV %	22.32	2.84	23.2	20.9	24.3	14	26.6	0.89	0.001	19.7 – 29.3
Total protein g/L	70.9	4.23	69.5	68	74	65	79	0.92	0.006	60 – 80
Albumin g/L	33.38	2.61	33	32	34	28	40	0.94	0.030	27 – 39
Creatinine mg/dL	1.26	0.286	1.2	1.0	1.4	0.8	1.8	0.93	0.014	0.6 – 1.8
Urea mg/dL	62.3	17.38	62.9	47.1	73	29	99	0.97	0.451	25 – 70
Sodium mmol/L	152.0	2.63	152.6	150.0	153.8	145.9	157.0	0.97	0.372	145 – 158

* Haematological reference intervals based on the results of the study of Granat et al. [9]; biochemical reference intervals according to a commercial veterinary laboratory from Warsaw in Poland (Lab-Wet); S.D.: standard deviation; Med.: median; 25th%: twenty fifth percentile; 75th%: seventy fifth percentile; Min.: minimal value; Max.: maximal value; W: W value in Shapiro–Wilk's W test; p: p value in Shapiro–Wilk's W test; Ref. int.: reference interval; T4: thyroxine concentration; RBC: red blood cell count; Hb: haemoglobin concentration; Hct:

haematocrit; MCV: mean corpuscular volume; RDW-CV: red blood cell distribution width - coefficient of variation.

Table S3. Statistics and results of Shapiro-Wilk W test in 46 hyperthyroid female cats.

	Mean	S.D.	Med.	25th%	75th%	Min.	Max.	W test result		Ref. int. *
								W	p	
Age in years	12.63	3.04	13	11	15	4	19	0.97	0.353	–
T4 nmol/L	110.6	47.6	94.5	73	143	61	236	0.86	0.000	10 – 30
RBC × 10^6/μL	9.59	1.89	9.75	8.22	10.6	4.72	13.4	0.98	0.748	6.7 – 11.2
Hb mmol/L	8.45	1.49	8.495	7.8	9.31	4.16	11.43	0.97	0.282	6.15 – 10.29
Hct L/L	0.417	0.069	0.42	0.39	0.47	0.23	0.56	0.97	0.304	0.29 – 0.51
MCV fL	44.12	4.88	44	40	47.4	32.8	56.6	0.99	0.980	34.4 – 49.3
RDW-CV %	22.27	2.72	23.25	20.4	24.1	13.4	27.5	0.90	0.000	19.7 – 29.3
Total protein g/L	72.32	4.7	72	69	77	64	80	0.96	0.095	60 – 80
Albumin g/L	35.28	2.77	35.5	34	37	28	40	0.96	0.151	27 – 39
Creatinine mg/dL	1.33	0.29	1.3	1.1	1.5	0.8	1.8	0.93	0.013	0.6 – 1.8
Urea mg/dL	67.5	19.45	71	50	81	26	98.3	0.96	0.197	25 – 70
Sodium mmol/L	152.0	3.131	152.2	149.4	154.5	145.6	157.1	0.96	0.237	145 – 158

* Haematological reference intervals based on the results of the study of Granat et al. [9]; biochemical reference intervals according to a commercial veterinary laboratory from Warsaw in Poland (Lab-Wet); S.D.: standard deviation; Med.: median; 25th%: twenty fifth percentile; 75th%: seventy fifth percentile; Min.: minimal value; Max.: maximal value; W: W value in Shapiro–Wilk's W test; p: p value in Shapiro–Wilk's W test; Ref. int.: reference interval; T4: thyroxine concentration; RBC: red blood cell count; Hb: haemoglobin concentration; Hct: haematocrit; MCV: mean corpuscular volume; RDW-CV: red blood cell distribution width - coefficient of variation.

Table S4. Results of Levene's test for homogeneity of variances in males and females (age, RBC count, Hb concentration, Hct, and MCV), and results of Student's t test comparing age, RBC count, Hb concentration, Hct and MCV between male and female cats.

Variable	M $\bar{x}$ ± S.D.	F $\bar{x}$ ± S.D.	t	t test p	F Levene	p Levene
Age in years	12.95 ± 2.89	12.63 ± 3.03	0.508	0.613	0.106	0.745
RBC × 10^6/μL	9.39 ± 1.58	9.59 ± 1.89	-0.533	0.595	0.654	0.421
Hb mmol/L	8.39 ± 1.31	8.45 ± 1.49	-0.213	0.832	0.156	0.694
Hct L/L	0.407 ± 0.054	0.417 ± 0.069	-0.768	0.445	1.701	0.196
MCV fL	43.74 ± 4.44	44.12 ± 4.88	-0.375	0.708	0.154	0.695

M $\bar{x}$: mean in males; F $\bar{x}$: mean in females; S.D.: standard deviation; t: a value of t in Student's t test; t test p: a value of p in Student's t test; F Levene: a value of F in Levene's test; p Levene: a value of p in Levene's test; RBC: red blood cell count; Hb: haemoglibin concentration; Hct: haematocrit; MCV: mean corpuscular volume.

Table S5. Correlations between erythrocyte parameters in 88 hyperthyroid cats.

Variable	RBC	Hb	Hct	MCV	RDW-CV
RBC	-	R: 0.812, p: 0.000	r: 0.799, p: 0.000	r:-0.572, p: 0.000	R: 0.002, p: 0.987
Hb		-	r: 0.879, p: 0.000	r: -0.172, p: 0.108	R: 0.017, p: 0.871
Hct			-	r: 0.019, p: 0.866	R: -0.150, p: 0.162
MCV				-	R: -0.163, p: 0.128
RDW-CV					-

RBC: red blood cell count; Hb: haemoglobin concentration; Hct: haematocrit; MCV: mean corpuscular volume; RDW-CV: red blood cell distribution width - coefficient of variation.

Table S6. Values of red blood cell count and mean corpuscular volume in the cats with recognized changes.

Cat No.	RBC × 10^6/μL	MCV fL	Changes in RBC
1	11.48	47.0	Erythrocytosis
7	11.77	40.0	Erythrocytosis
8	12.07	41.0	Erythrocytosis
13	12.06	43.0	Erythrocytosis
15	5.68	44.0	Anaemia
19	4.72	48.0	Anaemia
20	12.70	43.7	Erythrocytosis
29	13.62	34.0	Erythrocytosis, Microcytosis
32	7.29	53.0	Macrocytosis
35	6.04	56.6	Anaemia, Macrocytosis
45	11.49	39.1	Erythrocytosis
53	12.60	38.3	Erythrocytosis
56	11.70	33.5	Erythrocytosis, Microcytosis
62	13.40	37.6	Erythrocytosis
63	13.20	32.8	Erythrocytosis, Microcytosis
72	5.70	43.8	Anaemia
80	11.40	37.7	Erythrocytosis

Reference intervals for RBC × 10^6/μL (6.7 – 11.2), for MCV fL (34.4 – 49.3).

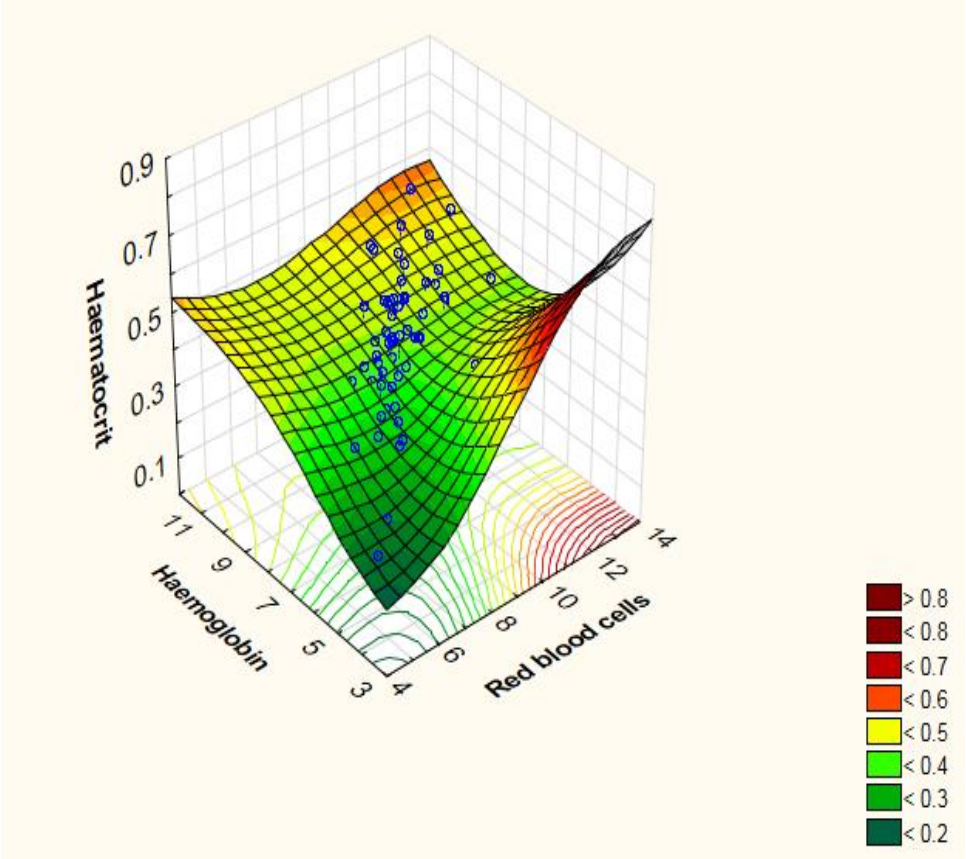

Figure S1. 3D surface function showing correlations between haematocrit, haemoglobin concentration and red blood cell count in 88 hyperthyroid cats (blue circles - cases; color scale from green to brown for haematocrit).
